# Mendelian randomization analysis identifies HLA‐A and AP2M1 as genetic biomarkers linked to immune–endocytic crosstalk in intervertebral disc degeneration

**DOI:** 10.1002/ccs3.70062

**Published:** 2026-02-14

**Authors:** Yukui Tian, Xue Bai, Nianrong Han, Cheng Wang, Junchang Liu

**Affiliations:** ^1^ Xinjiang Medical University/Xinjiang Medical University Affiliated Hospital of Traditional Chinese Medicine/Xinjiang Uyghur Autonomous Region Institute of Traditional Chinese Medicine Urumqi China; ^2^ Xinjiang Uyghur Autonomous Region Institute of Traditional Chinese Medicine Urumqi China; ^3^ Xinjiang Medical University Affiliated Hospital of Traditional Chinese Medicine Urumqi China

**Keywords:** AP2M1, biomarkers, endocytosis, HLA‐A, intervertebral disc degeneration

## Abstract

Intervertebral disc degeneration (IVDD) is a major contributor to chronic spinal disorders, yet the role of endocytosis in its pathogenesis remains incompletely understood. In this study, we systematically investigated endocytosis‐related genes associated with IVDD by integrating bulk transcriptome data, single‐cell RNA sequencing datasets, and Mendelian randomization (MR) analysis. Differential expression analyses identified six ERGs consistently dysregulated in IVDD, among which HLA‐A and AP2M1 exhibited significant causal associations with disease risk in MR analysis and were further validated in independent datasets. Functional enrichment and gene set enrichment analyses indicated that these genes were closely involved in immune‐related pathways, including natural killer cell‐mediated cytotoxicity and mammalian target of rapamycin signaling. Immune infiltration analysis revealed marked alterations in macrophages, activated CD4^+^ T cells, and eosinophils in IVDD tissues, with strong correlations between immune cell proportions and the expression of HLA‐A and AP2M1. In vitro experiments demonstrated that overexpression of HLA‐A or AP2M1 promoted nucleus pulposus cell proliferation, suppressed apoptosis, and enhanced endocytic activity, whereas in vivo overexpression alleviated disc degeneration in a rat model. Collectively, these findings identify HLA‐A and AP2M1 as potential biomarkers linking immune dysregulation and endocytic dysfunction in IVDD and provide new insights into the molecular mechanisms underlying disc degeneration.

## Graphical abstract

HLA‐A and AP2M1 are identified as key endocytosis‐related biomarkers that modulate immune–endocytic crosstalk via mTOR signaling to regulate cell survival, immune infiltration, and the progression of intervertebral disc degeneration.

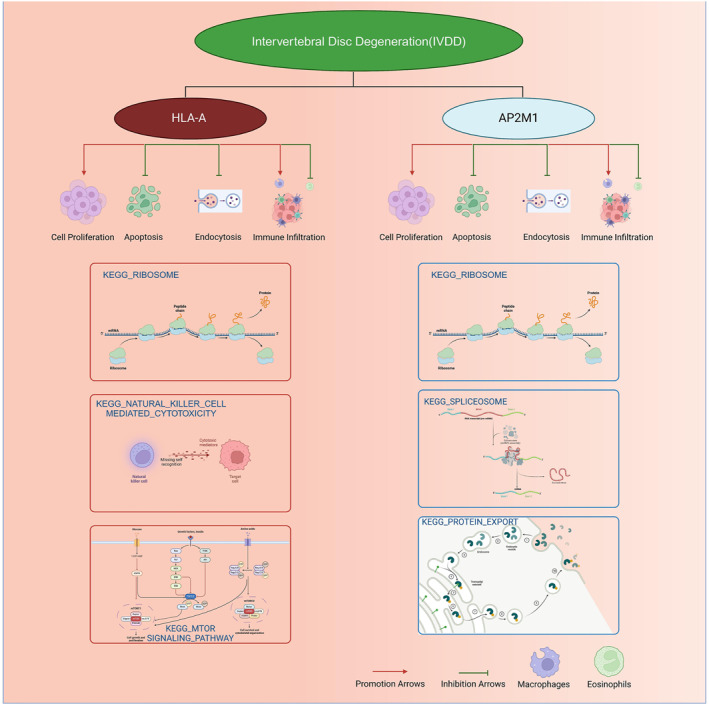

## 1 INTRODUCTION

Intervertebral disc degeneration (IVDD) is a major chronic condition in spinal surgery and plays a central role in the development of multiple degenerative spinal disorders.^1^ These diseases are often caused by pathological conditions, such as disc herniation, spinal stenosis, instability, spondylolisthesis, radiculopathy, scoliosis, and other abnormalities, which lead to acute or chronic pain in the cervical and lumbar regions.^2^, ^3^ Clinical symptoms usually emerge when the degenerated intervertebral disc (IVD) irritates or compresses adjacent nerve roots or the spinal cord, resulting in pain, motor deficits, or sensory dysfunction in the regions innervated by the affected nerves.^4^

Age‐related changes contribute significantly to disc degeneration. The number and metabolic activity of IVD cells, particularly nucleus pulposus (NP) cells, progressively decline with age, leading to reduced water content and gradual structural deterioration.^5^ The combined effects of water loss, proteoglycan depletion, and extracellular matrix (ECM) degradation weaken the mechanical properties of the disc and promote spinal instability.^6^, ^7^, ^8^ Clinically, IVDD is typically characterized by low back pain, radiculopathy, muscle weakness, and sensory disturbances.^9^ Multiple factors, including aging, genetic predisposition, mechanical loading, and lifestyle, influence the progression of degeneration.^5^

Recent findings indicate that a dual microRNA delivery strategy can promote disc regeneration by establishing an anti‐catabolic environment, with particularly notable effects in moderate degeneration, although further validation is needed.^10^ Recent studies have highlighted the critical roles of immune microenvironment alterations and metabolic imbalances in IVDD, enhancing our understanding of its pathophysiology.^9^, ^11^, ^12^ A more profound understanding of the mechanisms underlying IVDD is essential for the development of effective prevention and treatment strategies.

Endocytosis governs the selective internalization, processing, and intracellular transport of extracellular substances through the formation and trafficking of endocytic vesicles.^13^ Endocytic activity enables cells to regulate their internal environment by clearing excess molecules and metabolic waste.^14^ Under physiological conditions, endocytosis contributes to cellular homeostasis through dynamic regulation of the cell membrane and supports essential biological processes (BP), including cell growth, differentiation, and immune responses.^15^, ^16^, ^17^ Dysregulation of endocytosis has been linked to the development of several disorders, including neurodegenerative diseases, cancer, and metabolic dysfunction.^18^, ^19^ Recent evidence indicates that SPRY2 reduces FGFR1 expression by inhibiting endocytosis, thereby suppressing Fibroblast Growth Factor signaling and altering cell growth and cisplatin sensitivity.^20^ Given the fundamental role of endocytosis in cellular regulation, further investigation of its involvement in IVDD may reveal previously unrecognized pathogenic mechanisms.^21^, ^22^ Research studies focusing on endocytosis‐related genes (ERGs) in IVDD remain limited despite emerging evidence implicating endocytic dysfunction in degenerative disorders.^23^

IVD cells remove metabolic degradation products, including type II collagen and proteoglycans, through endocytosis to preserve ECM integrity. Impaired endocytosis causes the accumulation of ECM fragments and promotes the activation of proteases such as MMPs and ADAMTS enzymes, which further aggravates matrix metabolic imbalance. Recent studies have reported abnormal expression of specific endocytic regulators, including caveolin‐1, in NP cells from patients with IVDD, indicating that endocytic dysfunction may directly contribute to ECM metabolic disruption.^24^ The pathological microenvironment of IVDD is characterized by excessive secretion of inflammatory cytokines, including TNF‐α and IL‐1β.^25^ Endocytosis can inhibit downstream NF‐κB signaling by regulating the endocytic degradation of the IL‐1 receptor. Studies also found that inflammatory factors in NP cells can affect exosome secretion through the endocytic pathway, indirectly promoting the inflammatory response in neighboring cells. Excessive endocytic activity may accelerate the degradation of membrane receptors and initiate mitochondrial apoptotic signaling. Apoptotic cells are subsequently removed by phagocytic cells such as macrophages, which reduces secondary inflammation. IVD cells have been shown to function as effective phagocytes capable of internalizing latex beads and apoptotic cells.^26^

In recent years, the rapid development of bioinformatics technologies has provided effective tools for exploring the molecular mechanisms of complex diseases.^27^, ^28^ Large‐scale gene expression profiling enables the identification of differentially expressed genes (DEGs) associated with specific pathological conditions and facilitates detailed analysis of the signaling pathways involved. Biomarker screening, regulatory network construction, and immune infiltration analysis further support the systematic investigation of disease mechanisms.^28^, ^29^ Bioinformatics‐based approaches have been increasingly applied in studies of IVDD, revealing previously unrecognized molecular regulatory networks and offering new perspectives for understanding the pathogenesis of IVDD.^30^

Mendelian randomization (MR) is widely used to infer causal relationships between exposure factors and disease outcomes and has become an effective approach for identifying disease‐associated molecular biomarkers. Expression quantitative trait loci variants often serve as instrumental variables (IVs) to support causal inference. Rapid advances in single‐cell RNA sequencing (scRNA‐seq) technology enable high‐resolution characterization of gene expression at the single‐cell level, allowing detailed analysis of cellular heterogeneity and dynamic states. The integration of MR and scRNA‐seq provides deeper insight into disease mechanisms, as demonstrated by studies on Parkinson's disease, where synaptic dysfunction has been identified as an early pathological event and a potential target for early diagnosis and therapeutic intervention.^31^, ^32^, ^33^ Therefore, employing bioinformatics approaches to systematically study ERGs in IVDD holds significant scientific value and application potential.

Although research on endocytic dysfunction has progressed, the field remains in an early stage. Endocytosis governs the uptake and processing of extracellular substances and contributes to immune regulation, inflammatory signaling, and apoptosis.^26^, ^34^, ^35^ Analysis of endocytic mechanisms in IVDD may clarify key pathogenic processes and support the development of new therapeutic strategies. The present study integrated IVDD‐related datasets from the Gene Expression Omnibus (GEO) database with the ERG database for the first time. Using bioinformatics analyses, we identified potential biomarkers and validated their roles in IVDD through both in vitro and in vivo experiments. These findings offer theoretical support for the early diagnosis and personalized treatment of IVDD.

## 2 RESULTS

### 2.1 Biological functions of the six DE‐ERGs

Quality control of the two scRNA‐seq datasets was performed using the Seurat package (Figure S1A–D). The distributions of nFeature_RNA, nCount_RNA, and percent.mt indicated satisfactory cell quality after filtering. The top 2000 highly variable genes from each dataset were then identified based on standardized variance (Figure S1B,D).

**FIGURE 1:**
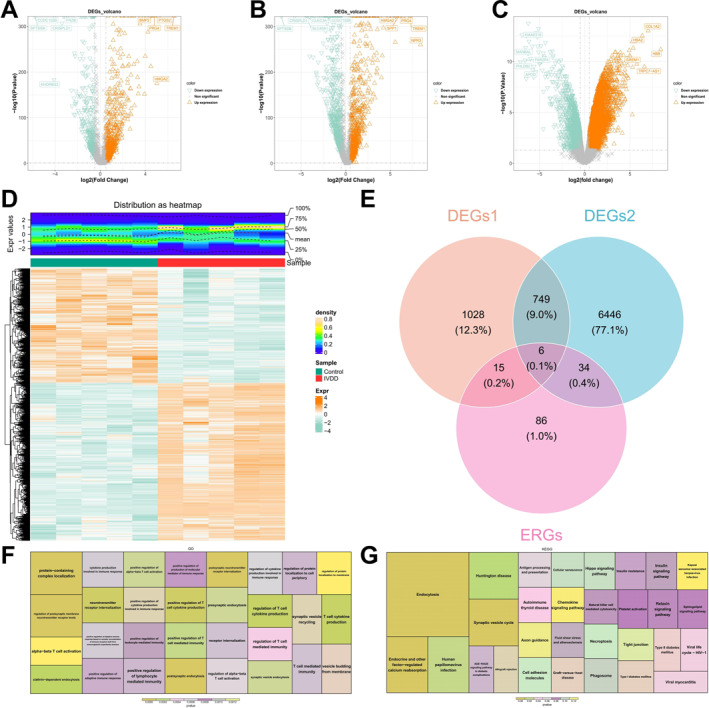
Identification of DEGs and differentially expressed endocytosis‐related genes, along with their functional enrichment analysis. (A, B) A total of 1142 DEGs were identified in AF cells, and 1517 DEGs were detected in NP cells. After merging and removing duplicates, 1798 unique DEGs were identified, designated as DEGs1; (C, D) In the GSE56081 dataset, 7235 DEGs were identified, named DEGs2; (E) An intersection analysis of DEGs1, DEGs2, and early response genes identified six differentially expressed ERGs; (F) GO enrichment analysis identified 568 enriched functional terms, at a significance threshold of FDR < 0.05, including 104 CC, 30 MF, and 434 BP. Significant terms include regulation of neurotransmitter receptor levels at the postsynaptic membrane, protein complex localization, and postsynaptic neurotransmitter receptor internalization; (G) KEGG enrichment analysis revealed 37 enriched pathways (FDR < 0.05), such as endocytosis, endocrine and other factor‐regulated calcium reabsorption, and synaptic vesicle cycle. AF, annulus fibrosus; BP, biological processes; DEGs, differentially expressed genes; FDR, false discovery rate; GO, gene ontology; KEGG, Kyoto encyclopedia of genes and genomes; MF, molecular functions.

After quality control (Figure S1A–D), 1142 DEGs were identified in annulus fibrosus (AF) cells and 1517 DEGs in NP cells. After merging and removing duplicates, 1798 DEGs were obtained and designated as DEGs1 (Figure 1A,B). In the GSE56081 dataset, 7235 DEGs were identified (Figure 1C,D).

An intersection analysis of DEGs1, DEGs2, and ERGs identified six DE‐ERGs (Figure 1E). Additionally, gene ontology (GO) enrichment analysis identified a total of 568 enriched functional terms, including 104 cellular components terms, 30 molecular functions (MF) terms, and 434 BP terms. Key enriched functions involved regulation of neurotransmitter receptor levels at the postsynaptic membrane, protein complex localization, and postsynaptic neurotransmitter receptor endocytosis (Figure 1F). Furthermore, Kyoto encyclopedia of genes and genomes (KEGG) enrichment analysis revealed 37 enriched pathways, such as endocytosis, endocrine and other factor‐regulated calcium reabsorption, and synaptic vesicle cycle (Figure 1G).

### 2.2 HLA‐A and AP2M1 identified as biomarkers

**FIGURE 2:**
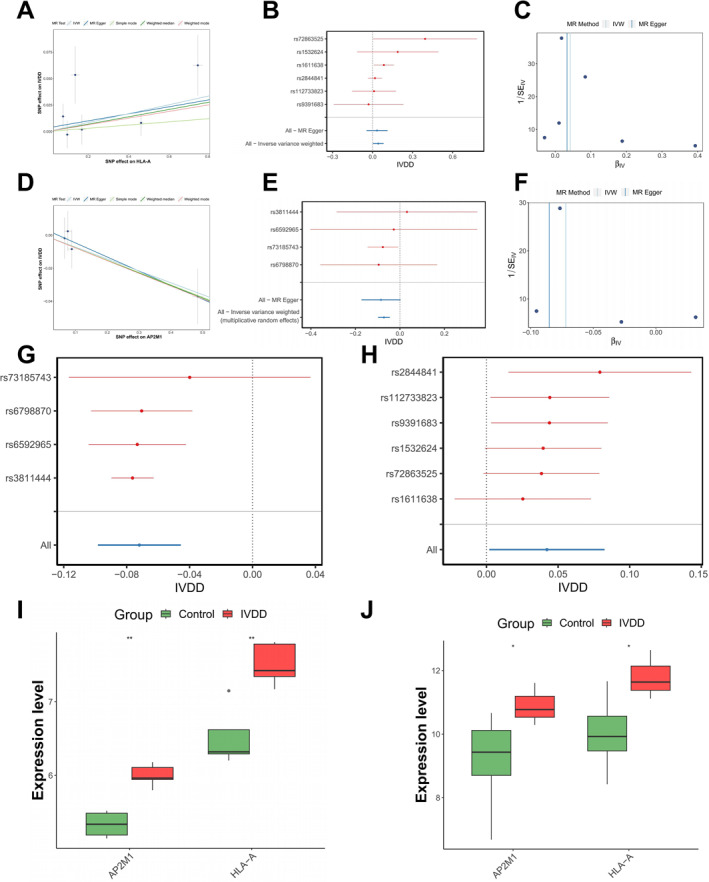
MR analysis and expression level comparison of HLA‐A and AP2M1 as candidate biomarkers for IVDD. (A) Scatter plot of HLA‐A in the MR analysis for IVDD, showing SNP effects and standard errors; (B) Forest plot of HLA‐A in the MR analysis for IVDD, displaying the effect estimates of different SNPs and their 95% confidence intervals; (C) Funnel plot of HLA‐A in the MR analysis for IVDD, assessing potential publication bias; (D) Scatter plot of AP2M1 in the MR analysis for IVDD, showing SNP effects and standard errors; (E) Forest plot of AP2M1 in the MR analysis for IVDD, displaying the effect estimates of different SNPs and their 95% confidence intervals; (F) Funnel plot of AP2M1 in the MR analysis for IVDD, assessing potential publication bias; (G, H) Sensitivity analysis results further confirming the reliability of the MR analysis, showing the effects of HLA‐A (G) and AP2M1 (H) on IVDD; (I, J) Comparison of gene expression levels of HLA‐A (I) and AP2M1 (J) between the IVDD and control groups using the GSE56081 training set (*n* = 10) and the GSE15227 validation set (*n* = 15), with significantly different expression levels (*p* < 0.05). *p < 0.05 between the two groups, **p < 0.01. MR analysis was performed using the TwoSampleMR package with GTEx (eQTL) as the exposure dataset and FinnGen (IVDD GWAS) as the outcome dataset. IVDD, intervertebral disc degeneration; MR, Mendelian randomization; SNP, single nucleotide polymorphism.

MR analysis identified two DE‐ERGs with causal relationships to IVDD. HLA‐A demonstrated a risk effect on IVDD (odds ratios [OR] = 1.04, 95% CI = 1.00–1.09), whereas AP2M1 exhibited a protective effect (OR = 0.93, 95% CI = 0.91–0.96) (Table 1). Scatter plots, forest plots, and funnel plots showed consistent results (Figure 2A–F). Specifically, in the two‐sample MR analysis, we assessed the causal relationships between the early response genes *HLA‐A* and *AP2M1* and IVDD. Scatter plots (Figure 2A,D) illustrated linear associations between single nucleotide polymorphism (SNP) effects on *HLA‐A*/*AP2M1* expression and IVDD. Regression trends generated by inverse‐variance weighted (IVW), MR‐Egger, and weighted median methods showed consistent patterns. Forest plots (Figure 2B,E) presented the causal effect estimates for individual SNPs with their confidence intervals, confirming *HLA‐A* as a risk factor and *AP2M1* as a protective factor. Funnel plots (Figure 2C,F) demonstrated symmetric distributions, indicating minimal pleiotropy and supporting the robustness of the analysis.

**TABLE 1:** MR analysis results evaluating the causal relationship between IVDD and exposure variables using different methods.

Outcome	Exposure	Method	*b*	SE	*p* value
Intervertebral disc disorders	ENSG00000161203	MR Egger	−0.08515845	0.044607366	0.196460898
Intervertebral disc disorders	ENSG00000161203	Weighted median	−0.07602533	0.032232094	0.018339858
Intervertebral disc disorders	ENSG00000161203	Inverse variance weighted (multiplicative random effects)	−0.07194219	0.01342758	8.42E‐08
Intervertebral disc disorders	ENSG00000161203	Simple mode	−0.075075651	0.072285057	0.375345107
Intervertebral disc disorders	ENSG00000161203	Weighted mode	−0.077028624	0.034807013	0.113780683
Intervertebral disc disorders	ENSG00000161203	MR Egger	0.033291742	0.040136395	0.453472412
Intervertebral disc disorders	ENSG00000161203	Weighted median	0.034782932	0.023935316	0.146166706
Intervertebral disc disorders	ENSG00000161203	Inverse variance weighted (fixed effects)	0.04219574	0.020533281	0.039879721
Intervertebral disc disorders	ENSG00000161203	Simple mode	0.015250941	0.042490254	0.734307714
Intervertebral disc disorders	ENSG00000161203	Weighted mode	0.031668106	0.024709748	0.256194218

Abbreviations: IVDD, intervertebral disc degeneration; MR, Mendelian randomization.

Sensitivity analysis confirmed the reliability of the MR findings (Tables S1 and S2, Figure 2G,H), and the Steiger test validated the accuracy of the causal analysis (*p* < 0.0001) (Table S3).

Based on the MR results, HLA‐A and AP2M1 were selected as candidate biomarkers for further analysis. Both genes showed consistent expression trends in the training dataset GSE56081 and the validation dataset GSE15227, with significant differences observed between IVDD and control samples (*p* < 0.05). These findings supported the selection of HLA‐A and AP2M1 as biomarkers for the present study (Figure 2I,J).

### 2.3 Functional analysis of biomarkers

**FIGURE 3:**
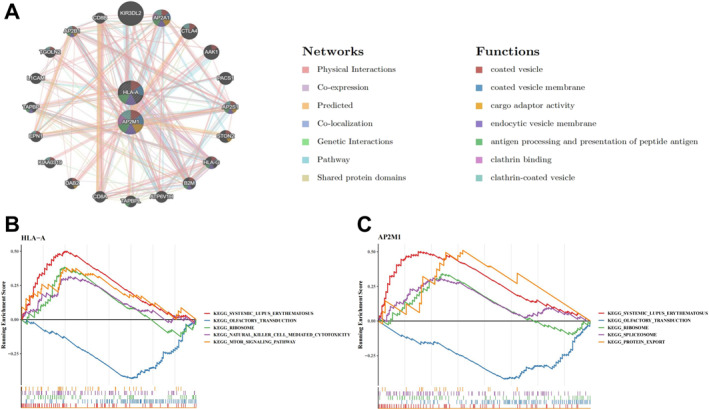
Functional analysis of biomarkers. (A) GGI network constructed based on the identified biomarkers, showing the top 20 genes interacting with HLA‐A and AP2M1, along with their relationships. The network includes 2 biomarkers, 20 interacting genes, and 383 interaction pairs; (B) GSEA pathway enrichment analysis of HLA‐A, showing its enrichment in the top 5 KEGG pathways. The top 5 enriched KEGG pathways include ribosome, natural killer cell‐mediated cytotoxicity, and the mTOR signaling pathway; (C) GSEA pathway enrichment analysis of AP2M1, showing its enrichment in the top 5 KEGG pathways. The significance threshold was set at FDR < 0.25 and *p* < 0.05. FDR, false discovery rate; GGI, gene‐gene interaction; GSEA, gene set enrichment analysis; KEGG, Kyoto encyclopedia of genes and genomes.

A gene–gene interaction (GGI) network was constructed using the two identified biomarkers. The network included the biomarkers, 20 interacting genes, and 383 interaction pairs organized by Score values (Figure 3A). The top functions enriched by false discovery rate (FDR) values included vesicle interior, vesicle membrane, and cargo adaptor activity, indicating functional relevance to vesicle trafficking and endocytic regulation.

Gene set enrichment analysis (GSEA) identified 21 KEGG pathways enriched for HLA‐A and 17 pathways enriched for AP2M1 (*p* < 0.05) (Figure 3B,C). The top enriched pathways for HLA‐A included ribosome, natural killer (NK) cell‐mediated cytotoxicity, and the mammalian target of rapamycin (mTOR) signaling pathway (*p* < 0.05).

### 2.4 High correlation between macrophages, eosinophils, and biomarkers

**FIGURE 4:**
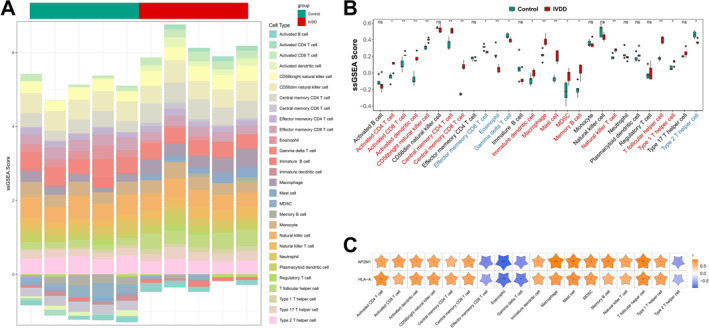
Correlation analysis between macrophages, eosinophils, and biomarkers. (A) Proportion of 28 immune cell types in IVDD and normal samples calculated using the ssGSEA algorithm (GSE56081 dataset); (B) Comparison of the proportions of 18 immune cell types between the control and IVDD groups, showing significant differences in activated CD4^+^ T cells, activated CD8^+^ T cells, activated dendritic cells, and others. Statistical analysis was performed using the Wilcoxon test. *p < 0.05, **p < 0.01, ***p < 0.001; (C) Correlation analysis between AP2M1 and macrophages and eosinophils, demonstrating the strongest positive correlation with macrophages (*r* = 0.9394, *p* < 0.001) and the strongest negative correlation with eosinophils (*r* = −0.9758, *p* < 0.001). *p < 0.05, **p < 0.01. IVDD, intervertebral disc degeneration.

In the GSE56081 dataset, the single‐sample GSEA (ssGSEA) algorithm was used to calculate the proportions of 28 immune cell types in IVDD and normal samples, as shown in Figure 4A. A comparison of the proportions of 18 immune cell types between the control and IVDD groups revealed significant differences in activated CD4^+^ T cells, activated CD8^+^ T cells, activated dendritic cells, etc. (Figure 4B).

Correlation analysis showed that AP2M1 had the highest positive correlation with macrophages (*r* = 0.9394, *p* < 0.001) and the highest negative correlation with eosinophils (*r* = −0.9758, *p* < 0.001) (Figure 4C).

### 2.5 TF‐biomarker‐miRNA regulatory network

**FIGURE 5:**
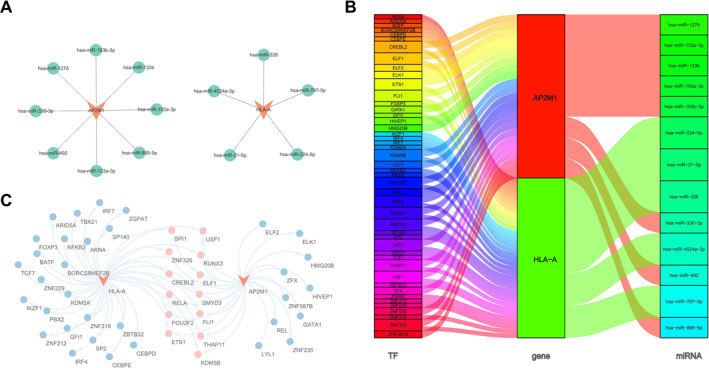
Correlation analysis between macrophages, eosinophils, and biomarkers. (A) miRNA‐biomarker regulatory network, showing 13 miRNAs predicted to target HLA‐A and AP2M1; (B) TF‐biomarker interaction network based on the ChEA3 database, including 2 biomarkers, 46 TFs, and 59 interaction pairs; (C) Prediction of 13 TFs targeting both HLA‐A and AP2M1. TF, transcription factor.

To elucidate the molecular regulatory mechanisms of the identified biomarkers, a microRNA–biomarker regulatory network was constructed. Thirteen microRNAs were predicted to target HLA‐A and AP2M1, including hsa‐miR‐1275, hsa‐miR‐326, and hsa‐miR‐133b (Figure 5A). The ChEA3 database was used to predict transcription factor (TF)‐biomarker relationships, generating a network comprising the two biomarkers, 46 TFs, and 59 interaction pairs (Figure 5B).

Notably, 13 TFs were predicted to target both biomarkers, including SPL1, USF1, and RUNX3 (Figure 5C). These findings were integrated into a comprehensive TF‐biomarker‐miRNA regulatory network, revealing potential regulatory pathways underlying the functions of the biomarkers.

### 2.6 The protective role of HLA‐A and AP2M1 in IVDD through the regulation of endocytosis and immune cell infiltration

Western blot (WB) analysis demonstrated that overexpression and silencing of HLA‐A and AP2M1 led to corresponding increases and decreases in their protein levels in NP cells and macrophage THP‐1 cells (Figure S2A,B).

**FIGURE 6:**
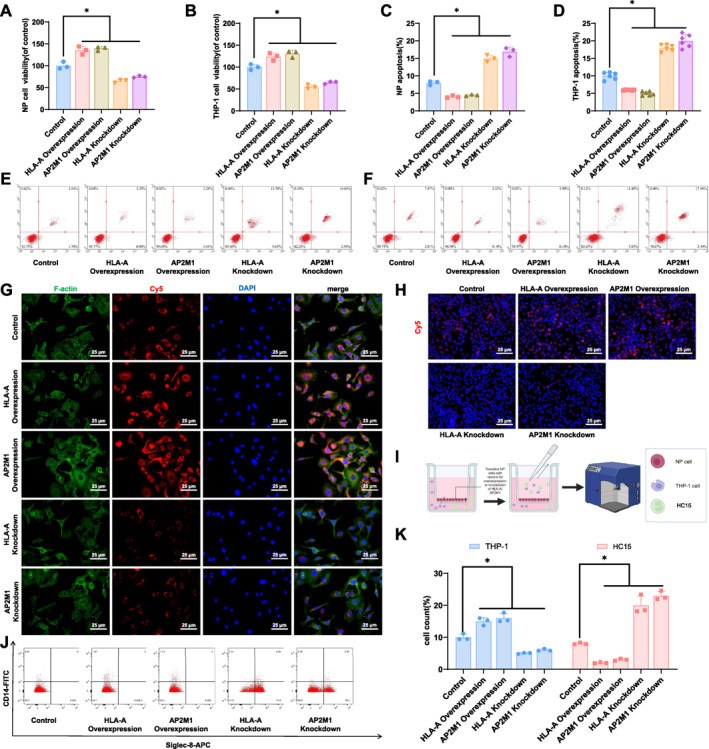
Functional analysis of HLA‐A and AP2M1 in IVDD. (A, B) CCK‐8 assays to detect the proliferation of NP cells and macrophage THP‐1 cells upon HLA‐A and AP2M1 overexpression; (C–F) Flow cytometry analysis of apoptosis in NP cells and THP‐1 cells after HLA‐A and AP2M1 overexpression; (G, H) Fluorescence intensity measurements of endocytic activity in NP cells and THP‐1 cells upon HLA‐A and AP2M1 overexpression; (I) Schematic diagram of the immune infiltration analysis workflow; (J, K) Flow cytometry analysis of immune cell infiltration, showing changes in macrophage and eosinophil proportions in NP and THP‐1 cells overexpressing HLA‐A and AP2M1. *p < 0.05. CCK‐8, cell counting kit‐8; IVDD, intervertebral disc degeneration; NP, nucleus pulposus.

The effects of HLA‐A and AP2M1 on cell viability were evaluated using the cell counting kit‐8 (CCK‐8) assay. Cell proliferation significantly increased in the HLA‐A overexpression and AP2M1 overexpression groups, whereas proliferation decreased in the HLA‐A knockdown and AP2M1 knockdown groups. Proliferation rates in both overexpression groups were higher than those in the control group, whereas knockdown of HLA‐A or AP2M1 resulted in markedly reduced proliferation (Figure 6A,B).

Flow cytometry was used to evaluate apoptosis in NP cells. Overexpression of HLA‐A and AP2M1 significantly reduced apoptosis, whereas knockdown of either gene markedly increased apoptosis (Figure 6C,E). Apoptosis rates were lower in both overexpression groups and higher in the knockdown groups compared with controls (*p* < 0.05) (Figure 6D,F).

Phagocytosis assays demonstrated that overexpression of HLA‐A and AP2M1 enhanced phagocytic activity in NP and THP‐1 cells, whereas knockdown of either gene significantly reduced phagocytosis. Both HLA‐A and AP2M1 overexpression groups exhibited increased phagocytic activity, whereas knockdown groups showed a marked decline (Figure 6G,H).

HL‐60 clone 15 (HC15) cells were used to generate eosinophil‐like cells through differentiation, a model widely applied in eosinophil research.^36^, ^37^ HC15 is a variant of the human promyelocytic leukemia cell line HL‐60, originally reported in the 1980s. It is generated by continuous culture of HL‐60 cells under mildly alkaline conditions (pH 7.6–7.8) for approximately 2 months. When stimulated with 0.5 mM sodium butyrate (SB) for 5–7 days, these cells can differentiate into eosinophil‐like cells.^38^, ^39^ In differentiated HC15 cells, the histone deacetylase inhibitor SB induces sustained acetylation of histones H3 and H4, leading to the expression of eosinophil‐associated features, such as CCAAT/enhancer‐binding transcription factors (C/EBPs), eosinophil major basic protein, and β7 integrin.^40^ Subsequent studies demonstrated that combined stimulation with SB and IL‐5 treatment enhances eosinophil‐like characteristics, particularly chemotactic behavior.^37^ The workflow for immune infiltration analysis is shown in Figure 6I. Overexpression of HLA‐A and AP2M1 significantly increased the proportion of macrophages and reduced the proportion of eosinophil‐like HC15 cells. Knockdown of HLA‐A and AP2M1 produced the opposite effect, with a significant increase in HC15 cells and a decrease in THP‐1 macrophages (Figure 6J). Compared with controls, macrophage proportions were higher and eosinophil proportions were lower in both overexpression groups (*p* < 0.05) (Figure 6K).

**FIGURE 7:**
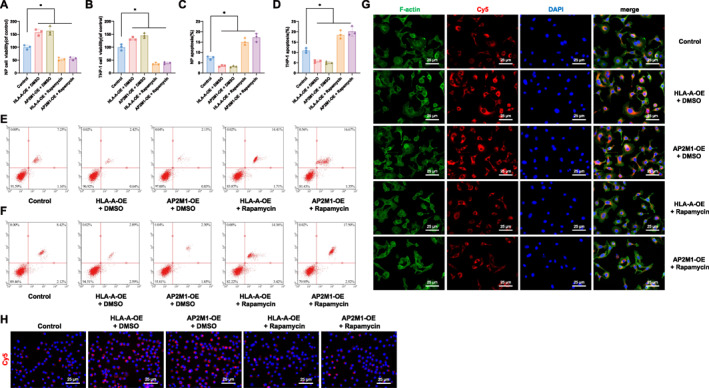
HLA‐A and AP2M1 regulate IVDD through the mTOR signaling pathway. (A, B) CCK‐8 assays assessing NP cell and THP‐1 macrophage proliferation in each group; (C–F) Flow cytometry analyses of apoptosis in NP cells and THP‐1 cells; (G, H) Fluorescence intensity measurements of endocytic activity in NP cells and THP‐1 cells across different treatment groups. *p < 0.05. CCK‐8, cell counting kit‐8; IVDD, intervertebral disc degeneration; mTOR, mammalian target of rapamycin; NP, nucleus pulposus.

Furthermore, in addition to overexpressing HLA‐A and AP2M1, we treated cells with the mTOR inhibitor Rapamycin. The results showed that Rapamycin significantly reversed the increased cell proliferation (Figure 7A,B), reduced apoptosis (Figure 7C–F), and enhanced endocytic activity (Figure 7G,H) induced by overexpression of HLA‐A or AP2M1. These findings indicate that HLA‐A and AP2M1 may regulate cell proliferation, apoptosis, and endocytic activity through the mTOR signaling pathway.

Taken together, these data suggest that overexpression of HLA‐A and AP2M1 in IVDD significantly promotes cell proliferation, inhibits apoptosis, enhances endocytic activity, and modulates macrophage and eosinophil infiltration.

### 2.7 In vivo validation of HLA‐A and AP2M1's protective role in IVDD through enhanced endocytosis

**FIGURE 8:**
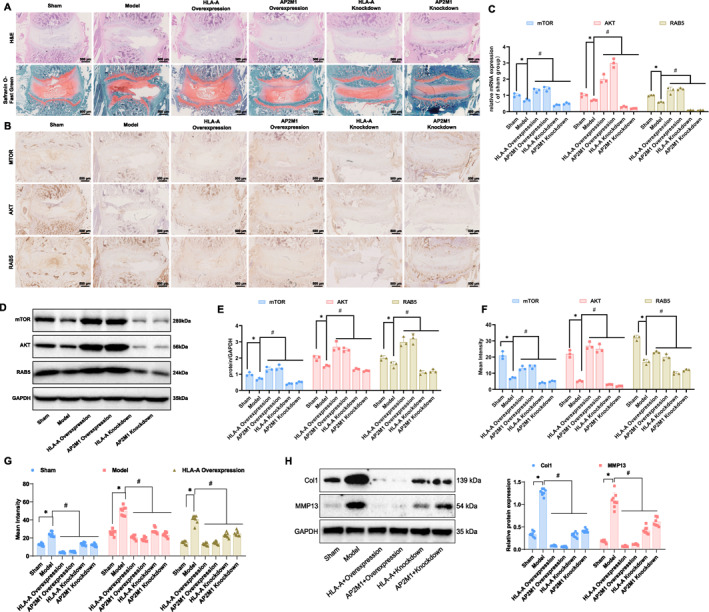
Effects of gene manipulation on IVDD in SD rats. (A) H&E staining and Safranin O‐Fast Green staining to assess the histological structure of rat intervertebral discs; (B) Immunohistochemical analysis of the impact of HLA‐A and AP2M1 on mTOR, AKT, and RAB5 protein expression; (C) Statistical analysis of mTOR, AKT, and RAB5 protein expression levels; (D, E) Western blot analysis of mTOR, AKT, and RAB5 protein expression in different groups; (F) RT‐qPCR analysis of mTOR, AKT, and RAB5 gene expression in different groups; (G) ELISA analysis of the levels of TNF‐α, IL‐1β, and IL‐6 in rat IVD tissues; (H) Western blot analysis of the expression of Col1 and MMP13 in rat IVD tissues in each group. *p < 0.05, ^#^ *p* < 0.01. ELISA, enzyme‐linked immunosorbent assay; H&E, hematoxylin and eosin; IVDD, intervertebral disc degeneration; mTOR, mammalian target of rapamycin; RT‐qPCR, Reverse Transcription Quantitative Polymerase Chain Reaction; SD, Sprague‐Dawley.

In the Sprague‐Dawley (SD) rat IVDD model, genetic manipulation of HLA‐A and AP2M1 significantly influenced disease progression. WB analysis demonstrated that protein levels of HLA‐A and AP2M1 were markedly reduced in IVD tissues of the model group compared with the sham group. Overexpression and knockdown treatments effectively increased and decreased their expression levels, respectively (Figure S2C). Histological analysis using hematoxylin and eosin (H&E) staining and Safranin O‐Fast Green staining showed that the IVD structure in the HLA‐A and AP2M1 overexpression groups maintained a more intact structural organization, with orderly cell alignment and preserved morphology in both the AF and NP regions. In contrast, the HLA‐A knockdown group and the AP2M1 knockdown group exhibited typical degenerative changes, including reduced cell numbers in the NP region and disrupted architecture in the AF (Figure 8A).

Immunohistochemistry analysis showed that expression levels of mechanistic target of mTOR, AKT, and RAB5 proteins were significantly increased in IVD tissues from the HLA‐A overexpression group and the AP2M1 overexpression group, indicating enhanced endocytic activity. The HLA‐A knockdown group and the AP2M1 knockdown group exhibited markedly reduced expression of these proteins (Figure 8B). Statistical analysis is shown in Figure 8C.

Molecular analyses further supported these findings. WB analysis revealed significantly higher expression of mTOR, AKT, and RAB5 proteins in the HLA‐A overexpression group and the AP2M1 overexpression group compared with controls, whereas knockdown of HLA‐A or AP2M1 significantly reduced the expression of these proteins (Figure 8D,E). Reverse Transcription Quantitative Polymerase Chain Reaction (RT‐qPCR) results showed a similar trend, with expression levels of mTOR, AKT, and RAB5 genes significantly increased in both overexpression groups and significantly decreased following knockdown of HLA‐A or AP2M1 (*p* < 0.05) (Figure 8F).

Enzyme‐linked immunosorbent assay (ELISA) showed that TNF‐α, IL‐1β, and IL‐6 levels in the IVD tissues of rats were significantly elevated in the model group compared with the Sham group. Overexpression of HLA‐A and AP2M1 significantly reduced the levels of these cytokines, whereas knockdown of either gene markedly increased cytokine concentrations (Figure 8G).

WB analysis showed a significant increase in the expression levels of ECM‐related proteins, collagen I (Col1), and MMP13, in the IVD tissues of rats in the model group compared to the sham group. Overexpression of HLA‐A and AP2M1 reduced the expression of HLA‐A and AP2M1 and Col1 and MMP13, whereas knockdown of both genes increased expression levels of these proteins (Figure 8H).

Immunofluorescence results further demonstrated the involvement of HLA‐A and AP2M1 in regulating immune cell infiltration. The proportions of macrophages were significantly higher and the proportions of eosinophils were markedly lower in the HLA‐A overexpression group and the AP2M1 overexpression group. Opposite trends were observed following knockdown, with increased eosinophil infiltration and reduced macrophage presence (Figure S2D,E).

In vivo experiments further confirmed the protective roles of these genes in the IVDD model, as evidenced by reduced degeneration and increased expression of proteins associated with endocytosis.

## 3 DISCUSSION

IVDD is a prevalent musculoskeletal disorder characterized by progressive structural and functional changes in the IVDs, often resulting in pain, stiffness, and reduced mobility in individuals exposed to aging or other risk factors.^41^ Recent research studies have shown that IVD cells can acquire a competent phagocytic state under specific in vitro conditions, enabling them to internalize extracellular material through endocytosis, which may contribute to disease progression.^26^ IVDD substantially reduces quality of life and imposes a growing economic burden on healthcare systems worldwide.^5^, ^42^ Given the potential involvement of endocytosis in IVDD progression, this study aimed to explore the role of ERGs in IVDD using transcriptome sequencing data from public databases and scRNA‐seq data. Bioinformatics analyses identified HLA‐A and AP2M1 as candidate biomarkers and revealed their regulatory associations with IVDD. The findings indicate that HLA‐A and AP2M1 may function as important diagnostic biomarkers and offer mechanistic insights into the progression of IVDD.

HLA‐A is a key component of the Major Histocompatibility Complex class I and plays an essential role in immune regulation. HLA‐A participates in T cell‐mediated cross‐presentation, a mechanism particularly prominent in dendritic cells. The process involves internalization of class I HLA proteins through early endosomes followed by antigen peptide loading within lysosomes, constituting a crucial part of the endocytic pathway.^43^ Previous studies have reported HLA‐A involvement in a variety of diseases. In cancer, altered HLA‐A expression contributes to immune evasion by tumor cells.^44^ In mucosal melanoma, HLA‐A has been evaluated as both a potential oncogenic driver and a therapeutic target.^45^ Studies have suggested that key genes such as HLA‐A, HMOX1, and JUN may influence the pathophysiology of post‐ICH depression by modulating immune mechanisms, including endocytosis, cell adhesion, and phagosome formation, thereby affecting the dopaminergic and serotonergic pathways.^46^ Additional evidence has shown that HLA class I antigens in the human leukemia T cell line HPB‐ALL undergo internalization in the absence of specific antibodies, suggesting participation in intercellular communication through endocytic processes.^47^ Another study reported that immune phagocytosis is mediated by both cytotoxic and noncytotoxic antibodies directed against HLA A, B, C, and DR antigens, highlighting the central role of HLA molecules in immune regulation.^48^ These findings indicate that HLA‐related genes contribute to immune responses and neurotransmitter‐associated processes implicated in the pathogenesis of psychiatric and immune‐mediated disorders. However, its specific role in IVDD has not been reported. This study is the first to identify significant expression of HLA‐A in IVDD, suggesting that HLA‐A may influence the initiation and progression of the disease.

AP2M1 is pivotal in endocytosis and vesicle transport.^49^ Abnormal expression of AP2M1 has been associated with neurodevelopmental disorders such as autism and Parkinson's disease,^50^ although its involvement in IVDD is limited. Previous studies examining the relationship between LRRK2 and endocytosis have shown that LRRK2 binds to and phosphorylates the μ1 subunit of AP2M1. AP2M1 functions as a core component of clathrin‐mediated endocytosis.^51^ This study also suggests that AP2M1 plays a significant role in the onset and progression of IVDD. AP2M1 has been identified as a target gene in various cancers, including hepatocellular carcinoma and acute myeloid leukemia.^52^, ^53^ Studies have shown that AP2M1 mediates the degradation of CLDN2 (claudin 2) through endocytosis and interaction with LC3, thereby reducing the permeability of intestinal epithelial tight junctions.^54^ Additionally, another bioinformatics‐based study revealed that AP2M1 is regulated by a circRNA‐miRNA‐mRNA interaction network, which may play a crucial role in the pathogenesis of Alzheimer's disease. These findings suggest that AP2M1 is closely involved in endocytosis and autophagy and may influence the progression of neurodegenerative diseases through complex RNA‐mediated regulatory mechanisms.

MR analysis revealed that HLA‐A is a risk factor for IVDD, whereas AP2M1 exerts a protective effect. The functional significance of HLA‐A and AP2M1 in regulating cell proliferation, apoptosis, and immune responses was further supported by GGI networks and GSEA. Their enrichment in pathways related to ribosomal activity, NK cell‐mediated cytotoxicity, and the mTOR signaling pathway was particularly notable. HLA‐A, a key component of MHC‐I molecules, presents pathogen‐derived peptides, including viral peptides during cytomegalovirus infection, enabling TCR‐like antibodies to recognize infected cells. This mechanism enhances the precision of immune responses and promotes the activation of NK cells and neutrophils to efficiently eliminate infected cells.^55^ Individual genotypes regulate HLA‐A expression levels and influence macrophage immune activity, highlighting the broad immunomodulatory potential of HLA‐A.^56^ AP2M1, an essential component of clathrin‐mediated endocytosis, plays a prominent role in shaping the tumor immune microenvironment. As a member of the mast cell signature model, AP2M1 has been shown to predict patient survival outcomes and responses to immunotherapy.^57^ Furthermore, AP2M1 can influence tumor immune evasion mechanisms by modulating the tumor immune microenvironment.^58^ These findings suggest that the endocytic functions of AP2M1 may regulate immune molecules and signaling pathways, thereby influencing the strength and durability of antitumor immunity. Although HLA‐A and AP2M1 differ in their primary functions, both exhibit strong associations with immune‐related mechanisms. HLA‐A directly modulates immune activity through antigen presentation and activation of T cells and NK cells. AP2M1 influences immune responses indirectly by regulating endocytosis and contributing to the architecture of the immune microenvironment.

Although HLA‐A and AP2M1 exhibit protective roles in IVDD, their ability to distinguish IVDD from other disorders remains uncertain and requires further investigation. The present study is based on analyses of previously published datasets, and heterogeneity within these datasets, as well as potential pleiotropy in MR analyses, may influence the reliability of our findings. Clinical evidence involving HLA‐A and AP2M1 is also limited, and additional studies are needed to evaluate their real‐world diagnostic and therapeutic value. The development of targeted interventions, as well as assessments of safety and potential side effects, requires further exploration. The present work provides preliminary evidence regarding the regulatory roles of HLA‐A and AP2M1 in IVDD, and future studies are needed to elucidate their specific molecular mechanisms. Although HLA‐A and AP2M1 show promise as biomarkers, their potential limitations should be acknowledged. Future research studies should comprehensively assess their clinical relevance in the prevention and treatment of IVDD to support the development of more effective therapeutic strategies.

Due to limitations in time and funding, the present study did not investigate the detailed molecular mechanisms underlying the functions of HLA‐A and AP2M1 in IVDD. Future research will focus on identifying the specific regulatory pathways through which HLA‐A and AP2M1 exert their effects. Planned work includes determining their positions within signaling pathways and characterizing their molecular interactions, including upstream regulators and downstream effector molecules. Additional analyses, such as protein–protein interaction and co‐expression network analyses, may be used to identify potential interacting partners and clarify their biological relevance. Although the validation dataset GSE15227 was derived from patients with femoral head necrosis, which differs from IVDD in disease background and may introduce biological heterogeneity, the expression trends of the key genes were consistent with those observed in the primary cohort. This finding suggests the presence of shared degenerative mechanisms across different skeletal disorders. Future studies will aim to define the specific roles of HLA‐A and AP2M1 in regulating endocytosis and immune cell infiltration. Additional investigations may evaluate their involvement in inflammatory signaling and cytokine production to deepen understanding of their contributions to IVDD. Ultimately, future studies should aim to elucidate the MF of HLA‐A and AP2M1 in IVDD, particularly how they influence disease progression through endocytosis and the immune microenvironment. Additional efforts are needed to validate these findings, explore their therapeutic potential at different disease stages, and develop early diagnostic tools and personalized treatment strategies, thereby advancing precision medicine in IVDD.

## 4 CONCLUSIONS

The present study identified HLA‐A and AP2M1 as potential biomarkers for IVDD through integrated bioinformatics analyses and in vitro and in vivo validation. These genes were found to participate in key BP, including ribosomal function, NK cell‐mediated cytotoxicity, and the mTOR signaling pathway. Immune infiltration analysis demonstrated strong associations between the biomarkers and macrophages and eosinophils, underscoring the importance of the immune microenvironment in IVDD. HLA‐A and AP2M1 offer promising molecular targets for early diagnosis and may support the development of personalized therapeutic strategies. The findings provide a foundation for future precision medicine research and clinical intervention, highlighting the potential translational value of these biomarkers in IVDD.

## 5 METHODS

### 5.1 Data sources

The IVDD‐related datasets GSE56081, GSE199866, and GSE15227 were obtained from the GEO (https://www.ncbi.nlm.nih.gov/geo/). The GSE56081 dataset (platform GPL15314) includes 5 IVDD and 5 control NP tissue samples. The GSE15227 dataset (platform GPL1352) consists of 12 IVDD samples with femoral head necrosis and 3 control samples from IVD tissue. Additionally, the single‐cell dataset GSE199866 (platform GPL20301) contains cell samples from 2 AF to 2 NP tissue samples.

The MR dataset finn‐b‐M13_INTERVERTEB was obtained from the Integrative Epidemiology Unit‐Open Genome Wide Association Studies (IEU‐Open GWAS) database (https://gwas.mrcieu.ac.uk/). The dataset includes 20,001 IVDD cases, 164,682 controls, and 16,380,337 SNPs from individuals of European ancestry without sample overlap. Using the keyword “Endocytosis,” 141 erg were extracted from the GSEA database.

### 5.2 Identification of DEGs1

Single‐cell sequencing data were analyzed using the Seurat package (version 5.0.0)^59^ to investigate cellular mechanisms involved in IVDD. Data filtering removed clusters with fewer than 3 cells, cells expressing fewer than 50 genes, cells with nFeature_RNA ≤200 or ≥7000, cells with nCount_RNA ≤200 or ≥50,000, and cells with percentage.mt ≥ 10%. Log normalization and vst transformation were applied to identify highly variable genes. The FindMarkers function in Seurat was used to analyze DEGs1 (|log_2_Fold Change| > 0.5, FDR <0.05, Minpct = 0.25). Finally, the results were visualized using the ggplot2 package (version 3.3.6)^60^ and pheatmap package (version 1.0.12).^61^

### 5.3 Identification and analysis of DE‐ERGs

Differential expression analysis was performed using the limma package (version 3.52.4)^62^ to generate DEGs2, with thresholds of |log_2_FC| > 0.5 and FDR <0.05. The DEGs2 results were visualized using ggplot2 and pheatmap. The ggvenn package (version 1.2.2)^63^ was then used to identify the intersection of DEGs1, DEGs2, and ERGs to obtain DE‐ERGs. GO and KEGG enrichment analyses were conducted using clusterProfiler (version 4.7.1.001)^64^ with *p* < 0.05. Treemaps of enriched terms were generated using the treemap package (version 2.4.4).^65^

### 5.4 MR analysis

MR analysis was conducted using the TwoSampleMR package (version 0.5.7)^66^ to validate potential biomarkers. MR analysis reduces reverse causality and confounding bias when three core assumptions are satisfied: strong correlation between IVs and exposure, no direct association between IVs and outcome, and independence from confounders.

First, the extract_instruments function was used to retrieve and filter IVs significantly associated with the exposure (*p* < 5 × 10^−8^) to ensure the inclusion of highly significant IVs. To address linkage disequilibrium issues, the clump_data function was applied with parameters set to *r* ^2^ = 0.001 and kb = 10,000 to remove any correlation between IVs. The harmonise_data function was then used to standardize effect sizes and scales to ensure consistency across datasets. IVs significantly associated with the outcome were excluded to reduce potential bias. MR analysis was performed using five methods: MR Egger, weighted median, IVW, simple mode, and weighted mode. IVW results were used as the primary reference, and OR were calculated, where OR > 1 indicated risk factors and OR < 1 indicated protective effects. Sensitivity analyses included heterogeneity testing (mr_heterogeneity), horizontal pleiotropy testing (mr_pleiotropy_test), and leave‐one‐out (mr_leaveoneout) analysis, with *p* > 0.05 indicating no significant heterogeneity or pleiotropy. Scatter plots, forest plots, and funnel plots were generated to visualize MR results. Steiger filtering was performed to assess causal direction, and genes with *p* < 0.05 and steiger dir = TRUE were considered candidate biomarkers. Finally, the expression of these candidate biomarkers was evaluated in the GSE56081 and GSE15227 datasets, and genes showing consistent and significant expression patterns were confirmed as biomarkers.

### 5.5 Functional annotation of biomarkers

After identifying the biomarkers, several analyses were performed to characterize their functional relevance. A GGI network was first constructed using GeneMANIA, and genes were ranked by Score. The top 20 interacting genes were selected as key nodes. Cytoscape (version 3.8.2)^67^ was then used to visualize the network. Spearman correlation analysis between each biomarker and all genes was performed using the psych package (version 2.3.6).^68^ GSEA was carried out using the clusterProfiler package (*p* < 0.05), with the human CP: KEGG gene set obtained from the msigdbr tool (version 7.5.1).^69^

### 5.6 Immune infiltration analysis

To compare immune cell infiltration between IVDD samples and controls, the ssGSEA algorithm in the GSVA package (version 1.48.3)^70^ was applied to quantify the proportions of 28 immune cell types in the GSE56081 dataset. Spearman correlation analysis using the psych package was performed to assess associations between biomarkers and immune cell types. Correlation plots were generated using the ggcor package (version 0.9.8.1).^71^ These analyses provided insight into immune‐related mechanisms associated with the biomarkers.

### 5.7 Construction of the TF‐biomarker‐miRNA regulatory network

To explore the regulatory mechanisms of the biomarkers, miRNA–biomarker interactions were predicted using the miRDB database (http://mirdb.org) and visualized in Cytoscape. TFs associated with the biomarkers were predicted using the ChEA3 database (https://maayanlab.cloud/chea3/) and integrated into a TF–biomarker network in Cytoscape. The predicted TFs and miRNAs were combined, and the TF–biomarker–miRNA regulatory network was visualized using the ggalluvial package (version 0.12.5).^72^

### 5.8 Cell culture and grouping

Human NP cells and THP‐1 macrophage cells were obtained from the American Type Culture Collection (ATCC) in 2023. THP‐1 is a human male‐derived monocyte cell line (RRID: CVCL_0006), originally isolated from the peripheral blood of a 1‐year‐old male patient with acute monocytic leukemia. NP cells were derived from human IVD NP tissue (Homo sapiens, NP, male), although no official RRID is available. All cell lines were authenticated by short tandem repeat profiling and confirmed to be mycoplasma‐free by PCR testing.

NP cells were cultured in Dulbecco's Modified Eagle Medium (DMEM; Gibco) containing 10% fetal bovine serum (FBS; Gibco) and 1% penicillin–streptomycin (Gibco), and maintained at 37°C with 5% CO_2_. All cell lines were used at passages 3–6. THP‐1 cells were cultured in RPMI‐1640 medium (Gibco) with 10% FBS under the same conditions. The experimental groups included: control, HLA‐A overexpression, AP2M1 overexpression, HLA‐A knockdown, AP2M1 knockdown, HLA‐A overexpression + Dimethyl Sulfoxide (DMSO) (HLA‐A‐OE + DMSO), AP2M1 overexpression + DMSO (AP2M1‐OE + DMSO), HLA‐A overexpression + Rapamycin (HLA‐A‐OE + Rapamycin), and AP2M1 overexpression + Rapamycin (AP2M1‐OE + Rapamycin). At 24 h after transfection, the corresponding groups were treated with 50 nM Rapamycin (HY‐10219, MCE) for 24 h, with equal volumes of DMSO used as controls.^73^

### 5.9 Cell transfection

Cell transfection was performed using Lipofectamine 3000 reagent (Invitrogen). Overexpression vectors for HLA‐A and AP2M1 and their corresponding siRNAs were introduced into NP and THP‐1 cells. Six hours after transfection, the medium was replaced with fresh culture medium, and subsequent experiments were conducted after 48 h.

### 5.10 CCK‐8 assay

Cell proliferation was assessed using the CCK‐8 kit (Dojindo). Transfected cells were seeded into 96‐well plates at a density of 5000 cells per well, with six replicates per group. At 24‐h intervals, 10 μL of CCK‐8 solution was added to each well and incubated at 37°C for 2 h. Absorbance at 450 nm was recorded using a microplate reader (Bio‐Rad). All experiments were independently repeated at least three times.

### 5.11 Flow cytometry for apoptosis and immune infiltration analysis

Apoptosis was assessed using the Annexin V‐FITC/PI Apoptosis Detection Kit (BD Biosciences). Transfected cells were collected, washed twice with Phosphate‐Buffered Saline (PBS), and resuspended in 100 μL of binding buffer. Annexin V‐FITC (5 μL) and PI (5 μL) were added, followed by incubation at ambient temperature in the dark for 15 min. Apoptosis was analyzed using a BD FACSCanto II flow cytometer (BD Biosciences), and data were processed with FlowJo software (Tree Star).

For immune cell infiltration analysis, Transwell chambers (Corning) were used. After transfection, NP cells were seeded in the upper chamber, and THP‐1 and HL‐60 clone 15 (HC15; ATCC‐CRL‐1964) cells (both obtained from ATCC) were co‐cultured in the lower chamber. Before co‐culture, HC15 cells were differentiated for 5 days using 0.5 mM SB (sc‐202341, Santa Cruz Biotechnology) and 10 ng/mL IL‐5 (NBP2‐34897, Biotechne GmbH, Wiesbaden Nordenstadt).^37^ The differentiated HC15 cells were then co‐incubated with THP‐1 cells at 37°C for 24 h. At the end of the culture period, cells from the upper chamber were collected and washed twice with PBS. CD14 antibody (BioLegend) and Siglec‐8 antibody (R&D Systems) were used to stain macrophages and eosinophils, respectively. The proportions of macrophages and eosinophils were analyzed using flow cytometry (BD FACSCanto II). All cell experiments were independently repeated at least three times.

### 5.12 Endocytosis assay

Endocytic activity was evaluated using Cy5‐labeled latex beads (Sigma‐Aldrich). Transfected cells were incubated with beads at a final concentration of 1 μg/mL for 1 h and then washed three times with PBS. Intracellular fluorescence signals were observed and recorded using a fluorescence microscope (Leica Microsystems). All cell experiments were independently repeated at least three times.

### 5.13 Animal model construction and grouping

An IVDD model was established in 8‐week‐old male SD rats (200–220 g, Charles River Laboratories). Rats were acclimated for 1 week under controlled conditions (22 ± 2°C, 12‐h light/dark cycle) with free access to food and water. A total of 60 rats were randomized into experimental groups using a random‐number table. Each rat was assigned a sequential number, a starting point was selected on the table, and consecutive numbers were transcribed to allocate animals into six groups (*n* = 8 per group). Rats were anesthetized using a intraperitoneal injection of 4% sodium pentobarbital (25 mg/kg, Sigma‐Aldrich). After anesthesia, the tail region was disinfected with 70% ethanol (Thermo Fisher Scientific). A 21G needle (BD Biosciences) was inserted into the center of the IVD between the seventh and tenth caudal vertebrae, rotated for 5 s, and held in place for 30 s to induce degeneration. Following puncture, rats were placed on a 37°C heating pad until recovery and then returned to their cages. In this experiment, adult male SD rats were randomly divided into six groups: the model group (*n* = 8), which underwent the IVDD modeling without any gene manipulation; the HLA‐A overexpression group (*n* = 8), in which adenoviral vectors carrying the HLA‐A plasmid were injected through the tail vein to construct the HLA‐A overexpression model; the AP2M1 overexpression group (*n* = 8), in which adenoviral vectors carrying the AP2M1 plasmid were injected through the tail vein to construct the AP2M1 overexpression model; the control group (*n* = 8), in which empty adenoviral vectors were injected through the tail vein; and the HLA‐A knockdown group (*n* = 8) and AP2M1 knockdown group (*n* = 8), in which respective HLA‐A or AP2M1 siRNA plasmids were injected through the tail vein for gene knockdown. Additionally, a sham operation group (*n* = 8) was included, in which only tail disinfection and puncture were performed without inducing IVDD or injecting any substances.

### 5.14 Histological analysis

IVD tissues were collected and fixed in 10% formalin (Sigma‐Aldrich) for 24 h, followed by dehydration, paraffin embedding, and sectioning at a thickness of 5 μm. H&E staining and Safranin O–Fast Green staining (Sigma‐Aldrich) were performed according to standard protocols. Structural changes in the AF and NP were examined using a microscope (Olympus). All assessments were performed in a blinded manner. Two independent investigators evaluated each section without knowledge of group assignments, and a third researcher compiled the data. Each group contained eight rats. The severity of disc degeneration was graded using the Thompson scoring system.^74^

### 5.15 Enzyme‐linked immunosorbent assay

TNF‐α, IL‐1β, and IL‐6 levels in rat IVD tissues were detected using TNF‐α ELISA kit (ab236712, Abcam), IL‐1β ELISA kit (255730, Abcam), and IL‐6 ELISA kit (ab234570, Abcam). Absorbance was recorded at 450 nm using an Epoch microplate reader (BioTek).^75^ Each group consisted of eight rats.

### 5.16 Immunohistochemistry analysis

Immunohistochemistry analysis was performed using the streptavidin–peroxidase method. Paraffin‐embedded sections were deparaffinized in xylene, rehydrated through graded ethanol, and treated with 3% H_2_O_2_ (Sigma‐Aldrich) to block endogenous peroxidase activity. Antigen retrieval was conducted in retrieval solution, followed by overnight incubation at 4°C with primary antibodies against mTOR (Cell Signaling Technology, 2983), AKT (Cell Signaling Technology, 9272), and RAB5 (Abcam, ab18211). HRP‐labeled goat antirabbit IgG (Cell Signaling Technology) was applied at 37°C for 1 h, followed by 3,3′‐Diaminobenzidine development and hematoxylin counterstaining. Images were captured using a microscope (Olympus), and staining intensity was quantified using ImageJ software (NIH). All evaluations were performed in a blinded manner by two independent investigators, and results were compiled by a supervisor after completion. Each group included eight animals.

### 5.17 WB analysis

WB was used to detect the expression levels of mTOR, AKT, RAB5, HLA‐A, AP2M1, Col1, and MMP13 proteins. Total protein was extracted from rat IVD tissue using Radioimmunoprecipitation Assay lysis buffer (Thermo Fisher Scientific) combined with a protease inhibitor cocktail (Roche). Protein concentration was determined using the Bicinchoninic Acid method (Pierce). A total of 20 μg of protein was separated by SDS‐PAGE (Bio‐Rad) and transferred onto a PVDF membrane (Millipore). The membrane was blocked with 5% skim milk for 1 h at ambient temperature. The following primary antibodies were used: mTOR (Cell Signaling Technology, Cat. No. 2983), AKT (Cell Signaling Technology, Cat. No. 9272), RAB5 (Abcam, Cat. No. ab18211), HLA‐A (Abcam, Cat. No. ab246691), AP2M1 (Abcam, Cat. No. ab75995), Col1 (Abcam, Cat. No. ab308221), MMP13 (Abcam, Cat. No. ab39012), and GAPDH (Cell Signaling Technology, No. 2118), with overnight incubation at 4°C. The secondary antibodies, HRP‐labeled goat antirabbit IgG or goat antimouse IgG (Cell Signaling Technology), were used. Protein bands were visualized using ECL reagent (Thermo Fisher Scientific) and captured with the Chemidoc XRS + system (Bio‐Rad). Band intensity was analyzed using Image Lab software (Bio‐Rad). Each group consisted of eight rats.

### 5.18 RT‐qPCR

RT‐qPCR was performed to evaluate mTOR, AKT, and RAB5 gene expression. Total RNA was isolated from rat IVD tissue using TRIzol reagent (Invitrogen). Complementary DNA was synthesized using a reverse transcription kit (Takara). qPCR was performed using SYBR Green Master Mix (Applied Biosystems) under the following cycling conditions: 95°C for 10 min, followed by 40 cycles at 95°C for 15 s and 60°C for 1 min. Primers were synthesized by Sangon Biotech, and the sequences are listed in Table 2. qPCR was conducted on the 7500 Fast Real‐Time PCR System (Applied Biosystems). β‐actin served as the internal reference, and data were analyzed using the 2^−ΔΔCt^ method. Each group consisted of eight rats.

**TABLE 2:** PCR primer sequences.

Gene	Primer type	Sequence (5'→3')
HLA‐A	Forward	CCGTCCAGAGGATGTATGGC
HLA‐A	Reverse	AAGAGCGCAGGTCCTCTTTC
AP2M1	Forward	AGATGACATCGGGAGGAACG
AP2M1	Reverse	GGACCGCTTAACGTGGAAGA
Mtor	Forward	GCAATGGGCACGAGTTTGTT
Mtor	Reverse	AGTGTGTTCACCAGGCCAAA
Akt1	Forward	GAAGGAGAAGGCCACAGGTC
Akt1	Reverse	TTCTGCAGGACACGGTTCTC
Rab5a	Forward	CGACGACGAGGAGGAAGAAG
Rab5a	Reverse	ACTCGCTGCTTCTCACACTG

### 5.19 Immunofluorescence assay

Immunofluorescence staining was performed to assess the involvement of HLA‐A and AP2M1 in macrophage and eosinophil infiltration. Tissue sections were fixed with 4% paraformaldehyde (Sigma‐Aldrich) for 30 min and permeabilized with 0.3% Triton X‐100 (Sigma‐Aldrich) for 10 min. Sections were then blocked with PBS containing 10% goat serum (Gibco) for 1 h. Primary antibodies against CD14 (1:200, Abcam) and Siglec‐8 (1:200, BioLegend) were applied for overnight incubation at 4°C. The next day, Alexa Fluor 488– and Alexa Fluor 594–conjugated secondary antibodies (1:500, Invitrogen) were incubated for 1 h at room temperature in the dark. Nuclei were stained with DAPI (1 μg/mL, Thermo Fisher Scientific). Images were acquired using a Leica TCS SP8 confocal microscope (Leica Microsystems), and macrophage and eosinophil proportions were quantified using ImageJ software (NIH). All evaluations were performed in a blinded manner by two independent investigators, and data were compiled by a designated supervisor after analysis. Each animal group contained eight rats. All cell‐based experiments were independently repeated at least three times.

### 5.20 Statistical analysis

All experimental data were analyzed using GraphPad Prism 8.0 software (GraphPad Software). Data are presented as the mean ± standard error of the mean (mean ± SEM). Before hypothesis testing, data distribution was assessed using the Shapiro–Wilk test for normality, and variance homogeneity was evaluated using Levene's test. For normally distributed data with equal variances, differences between two groups were analyzed using unpaired two‐tailed Student's *t*‐tests, whereas comparisons among multiple groups were performed using one‐way ANOVA followed by Bonferroni post‐hoc correction. For non‐normally distributed data or unequal variances, the Mann–Whitney *U* test or Kruskal–Wallis test with FDR correction was applied. Statistical analyses for the bioinformatics section were conducted using R software (version 4.2.1) with multiple testing corrections applied using the Benjamini–Hochberg method where appropriate. Unless otherwise stated, an adjusted *p*‐value (adj. *p*) < 0.05 or *p <* 0.05 was considered statistically significant.

## AUTHOR CONTRIBUTIONS

Yukui Tian and Xue Bai conceived and designed the study. Nianrong Han, Cheng Wang, and Junchang Liu performed the experiments. Yukui Tian and Xue Bai analyzed the data. Yukui Tian and Junchang Liu wrote the manuscript. All authors reviewed and approved the final version of the manuscript.

## CONFLICT OF INTEREST STATEMENT

The authors declare no conflicts of interest.

## ETHICS STATEMENT

All animal experiments were approved by the Animal Ethics Committee of Xinjiang Medical University (IACUC‐20250213‐01).

## Supporting information

Supporting Information S1

## Data Availability

The authors confirm that the data supporting the findings of this study are available within the article and its Supporting Information S1.
