# Supplementary material for: Mendelian randomization analysis identifies HLA‐A and AP2M1 as genetic biomarkers linked to immune–endocytic crosstalk in intervertebral disc degeneration
Source: J Cell Commun Signal. 2026 Feb 14;20(1):e70062. doi: 10.1002/ccs3.70062 (PMC12906310; doi:10.1002/ccs3.70062)
Supplement: Supplementary file 1 — Supporting Information S1 [file CCS3-20-e70062-s001.docx]

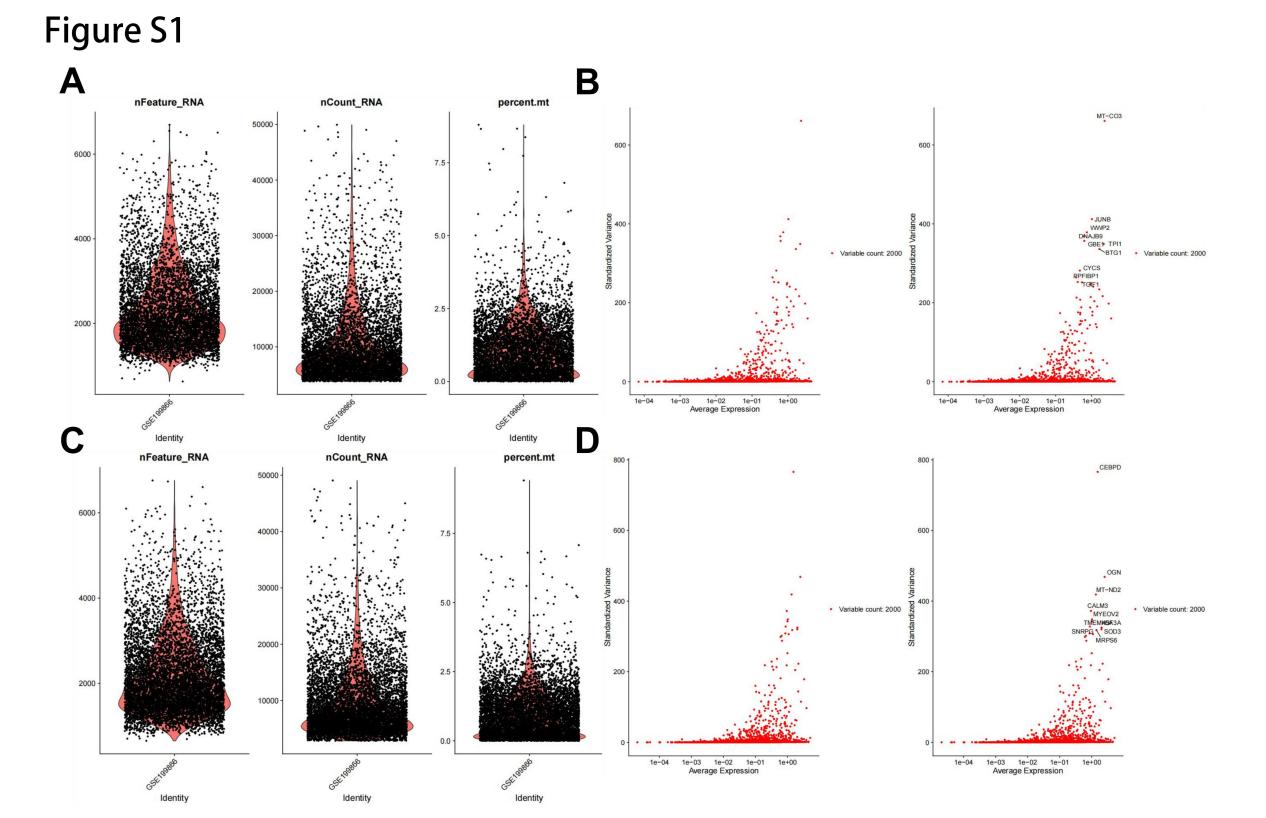


**Figure S1. Gene expression and differential gene analysis.**

Note: (A&C) The quality control results of single-cell samples. The violin plots display nFeature_RNA (number of detected genes), nCount_RNA (UMI counts), and percent.mt (percentage of mitochondrial genes) for each sample. Each dataset contains 12 samples (6 IVDD and 6 normal). Preprocessing was performed using the Seurat package, with filtering criteria of nFeature_RNA between 200–5,000 and percent.mt < 20%; (B&D) The identification of highly variable genes. The x-axis represents the mean expression of each gene, and the y-axis represents the standardized variance. Red dots indicate highly variable genes, which are typically used for downstream PCA dimensionality reduction. The top 2,000 highly variable genes were selected for each dataset.


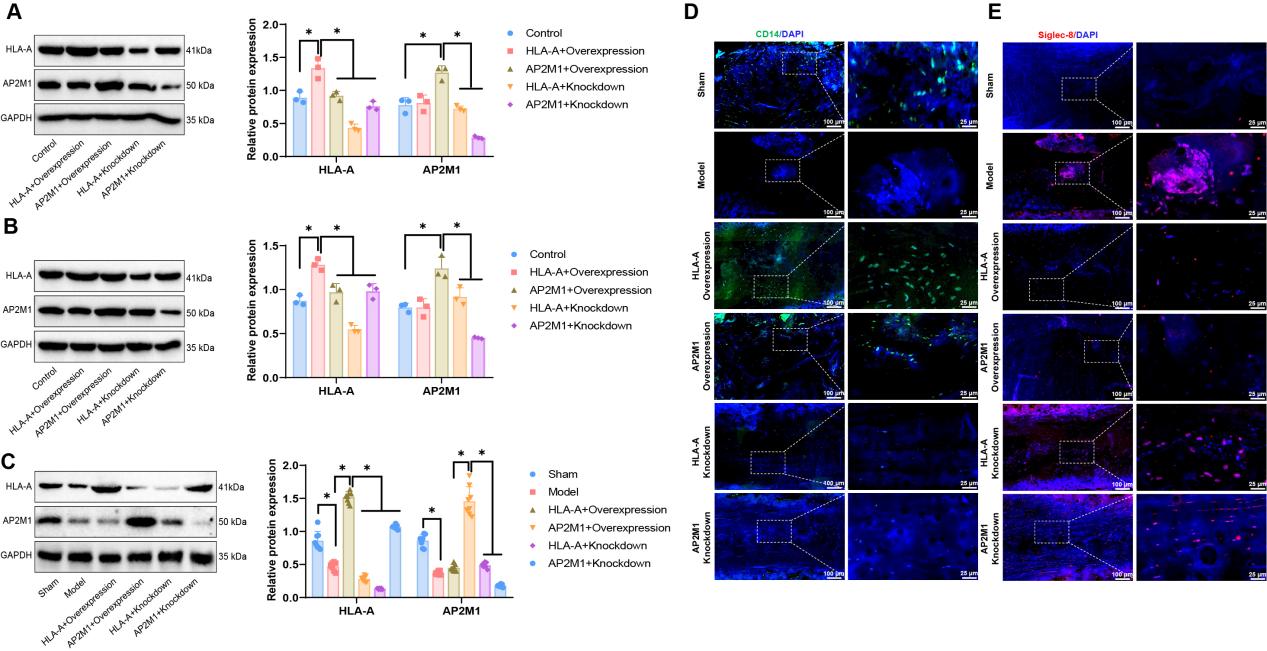


**Figure S2. Efficiency of HLA-A and AP2M1 overexpression and silencing and their impact on immune cell infiltration.**

Note: (A) Western blot detected the protein expression levels of HLA-A and AP2M1 in NP cells; (B) Western blot detected the protein expression levels of HLA-A and AP2M1 in macrophage THP-1 cells; (C) Western blot detected the protein expression levels of HLA-A and AP2M1 in rat IVD tissues; (D-E) Immunofluorescence staining analysis showed changes in the proportions of macrophages and eosinophils in frozen tissue sections.

**Table S1. MR Analysis Results for Identifying Candidate Biomarkers of IVDD.**

| **outcome** | **exposure** | **method** | **Q** | **Q_df** | **Q_pval** |
| --- | --- | --- | --- | --- | --- |
| Intervertebral disc disorders | ENSG00000206503 | MR Egger | 6.365437205 | 4 | 0.173468859 |
| Intervertebral disc disordersid | ENSG00000206503 | Inverse variance weighted | 6.499656805 | 5 | 0.260587783 |
| Intervertebral disc disorders | ENSG00000206503 | MR Egger | 0.329096858 | 2 | 0.848276675 |
| Intervertebral disc disorders | ENSG00000206503 | Inverse variance weighted | 0.515006551 | 3 | 0.915582383 |

**Table S2. Egger Regression Results for Evaluating Causal Relationships.**

| **outcome** | **exposure** | **egger_intercept** | **se** | **pval** |
| --- | --- | --- | --- | --- |
| Intervertebral disc disorders | ENSG00000206503 | 0.003420597 | 0.011778175 | 0.785930769 |
| Intervertebral disc disorders | ENSG00000206503 | 0.00384344 | 0.008913927 | 0.70836818 |

**Table S3. Steiger Analysis Results for Evaluating the Correct Direction of Causal Relationships.**

| **exposure** | **outcome** | **snp_r2.exposure** | **snp_r2.outcome** | **correct_causal_direction** | **steiger_pval** |
| --- | --- | --- | --- | --- | --- |
| ENSG00000161203 | Intervertebral disc disorders | 0.033884498 | 2.96E-05 | TRUE | 1.07E-192 |
| ENSG00000206503 | Intervertebral disc disorders | 0.086110029 | 5.81E-05 | TRUE | 0 |
